# Editorial: Community series in SIRT family in endocrinology

**DOI:** 10.3389/fendo.2023.1163226

**Published:** 2023-03-16

**Authors:** Yuxuan Hou, Ying Cheng, Ye Tian, Yang Yang

**Affiliations:** ^1^ Key Laboratory of Resource Biology and Biotechnology in Western China, Ministry of Education, Faculty of Life Sciences and Medicine, Northwest University, Xi’an, China; ^2^ Deparment of Neurology, Xi’an No. 3 Hospital, The Affiliated Hospital of Northwest University, Faculty of Life Sciences and Medicine, Northwest University, Xi’an, China

**Keywords:** editorial, SIRT family, endocrinology, chronic kidney disease, diabetes mellitus, obesity, hepatic steatosis, bone metabolism

The sirtuin (SIRT) family, which belongs to the class III histone deacetylases, comprises seven members (SIRT 1-7) that are localized in different subcellular compartments of the mammals. These seven members share a highly conserved NAD^+^ binding domain and a catalytic functional domain. Through its deacetylation function, the SIRT family regulates various biological processes, such as endocrinology, metabolism, and autophagy. Hence, SIRTs are major regulators of homeostasis.

The endocrine system coordinates physiological conditions and behavior, as well as the response to social environment, which is important for homeostasis. The SIRT family can regulate a range of endocrine signaling pathways, including nuclear factor-κB (NF-κB), peroxisome proliferator-activated receptor γ (PPARγ), signal transducer and activator of transcription 3 (STAT3), and forkhead-box transcription factors (FOXO). SIRT1 activation inhibits NF-κB by deacetylating the p65 subunit of the NF-κB complex to induce mitochondrial dysfunction, further drive caspase3-mediated cancer cell pyroptosis (1). In adipose tissues, SIRT1 activation interacts with PPARγ and increases the level of deacetylation of PPARγ, which promotes tissue remodeling and thermogenesis (2). However, SIRT3 knockout severely impairs insulin-stimulated muscle glucose uptake, which further exacerbates insulin resistance (3). These evidences indicate that dysregulated SIRTs activity is associated with a wide spectrum of diseases and targeting SIRT1 has promising applications in the treatment of endocrine diseases, which are the focus of this research topic.

The Research Topic covers the themes of versatile endocrine-related diseases, including chronic kidney disease, diabetes mellitus, obesity, hepatic steatosis, and bone metabolism. As thoroughly reviewed by Bian and Ren, the SIRT family regulates the pathophysiology of diabetic kidney disease, mesangial cell proliferation and hypertrophy, podocytes apoptosis, proximal tubular glucose metabolism, and renal tubular injury *via* multiple signaling pathway targets (PGC1α, NF-κB, FoxO1, FoxO3a, TGFβ1, and AMPK), epigenetics of deacetylation and dephosphorylation, or mitochondrial function (Sirtuin Family and Diabetic Kidney Disease). The author also summarized the currently used gene-specific expression animals, disease models, as well as SIRT-related activators and antagonists, which may be useful for the design of further mechanism researches. SIRT1 is the most prominent and extensively studied member of the family. Yan et al. provided an overview regarding the association between the increasing level of SIRT1 and renal protection, and focused their attention on chronic kidney disease and its complications (Sirtuin 1 in Chronic Kidney Disease and Therapeutic Potential of Targeting Sirtuin 1). Moreover, Kim et al. introduced the crosstalk between SIRT1 and autophagy as well as the effects of SIRT1-mediated autophagy in obesity, diabetes mellitus, diabetic cardiomyopathy, and hepatic steatosis in detail (SIRT1 and Autophagy: Implications in Endocrine Disorders). As their described, SIRT1 is involved in various steps of autophagy, including initiation, elongation, maturation, fusion, and degradation, which suggests that dysregulation of SIRT1-mediated autophagy may participate in the development of endocrine disorders. They also evaluated potential therapeutic interventions targeting SIRT1, such as resveratrol and NAD supplements. In addition to the above diseases, SIRT1 regulates bone mass and architecture. Artsi et al. aimed to elucidate the potential mechanism of sex differences in SIRT1 skeletal effects. Compared with male mice, they found that female mice had a higher bone SIRT1 level, while a dramatic decline with aging. Adult SIRT1 haplo-insufficiency mice displayed severe cortical bone deterioration, highlighting a potential therapeutic target of SIRT1 for ameliorating age-related cortical bone deterioration of females (SIRT1 haplo-insufficiency results in reduced cortical bone thickness, increased porosity and decreased estrogen receptor alpha in bone in adult 129/Sv female mice).

In summary, we hope that this research topic provides the reader with in depth understanding in SIRT family biology, signaling pathways, and activators, in particular, its effects on endocrinology and related diseases. We would like to thank the authors and reviewers for their excellent contributions, and their efforts in understanding this topic.

## Author contributions

All authors listed have made a substantial, direct and intellectual contribution to the work, and approved it for publication.
